# Differential Changes in QTc Duration during In-Hospital Haloperidol Use

**DOI:** 10.1371/journal.pone.0023728

**Published:** 2011-09-22

**Authors:** Marieke T. Blom, Abdennasser Bardai, Barbara C. van Munster, Mei-Ing Nieuwland, Hendrik de Jong, Daniel A. van Hoeijen, Anne M. Spanjaart, Anthonius de Boer, Sophia E. de Rooij, Hanno L. Tan

**Affiliations:** 1 Heart Failure Research Center, Department of Cardiology, Academic Medical Center, University of Amsterdam, Amsterdam, The Netherlands; 2 Department of Clinical Epidemiology, Biostatistics and Bioinformatics, Academic Medical Centre, University of Amsterdam, Amsterdam, The Netherlands; 3 Department of Internal Medicine and Geriatrics, Academic Medical Centre, University of Amsterdam, Amsterdam, The Netherlands; 4 Division of Pharmacoepidemiology and Clinical Pharmacology, Utrecht Institute for Pharmaceutical Sciences, Utrecht University, Utrecht, The Netherlands; Heart Center Munich, Germany

## Abstract

**Aims:**

To evaluate changes in QT duration during low-dose haloperidol use, and determine associations between clinical variables and potentially dangerous QT prolongation.

**Methods:**

In a retrospective cohort study in a tertiary university teaching hospital in The Netherlands, all 1788 patients receiving haloperidol between 2005 and 2007 were studied; ninety-seven were suitable for final analysis. Rate-corrected QT duration (QTc) was measured before, during and after haloperidol use. Clinical variables before haloperidol use and at the time of each ECG recording were retrieved from hospital charts. Mixed model analysis was used to estimate changes in QT duration. Risk factors for potentially dangerous QT prolongation were estimated by logistic regression analysis.

**Results:**

Patients with normal before-haloperidol QTc duration (male ≤430 ms, female ≤450 ms) had a significant increase in QTc duration of 23 ms during haloperidol use; twenty-three percent of patients rose to abnormal levels (male ≥450 ms, female ≥470 ms). In contrast, a significant decrease occurred in patients with borderline (male 430–450 ms, female 450–470 ms) or abnormal before-haloperidol QTc duration (15 ms and 46 ms, respectively); twenty-three percent of patients in the borderline group, and only 9% of patients in the abnormal group obtained abnormal levels. Potentially dangerous QTc prolongation was independently associated with surgery before haloperidol use (OR_adj_ 34.9, p = 0.009) and before-haloperidol QTc duration (OR_adj_ 0.94, p = 0.004).

**Conclusion:**

QTc duration during haloperidol use changes differentially, increasing in patients with normal before-haloperidol QTc duration, but decreasing in patients with prolonged before-haloperidol QTc duration. Shorter before-haloperidol QTc duration and surgery before haloperidol use predict potentially dangerous QTc prolongation.

## Introduction

Haloperidol has for many years been a widely prescribed drug for the treatment of agitation, delirium, acute and chronic psychoses. Haloperidol is a butyrophenon antipsychotic that reduces psychomotor disturbances such as agitation and hallucinations by blocking both dopamine D_2_ and α_1_-adrenergic receptors, causing an antidopaminergic effect in the mesocortex and limbic system of the human brain. Although haloperidol is effective in clinical practice, it has been associated with QTc prolongation on the ECG [1], [2], [3], [4], [5], [6]. As QT prolongation is an established risk factor for potentially life-threatening cardiac ventricular arrhythmias of the type Torsade de Pointes [7], [8], [9], [10], clinicians are advised to keep a keen eye on (cardiac) medical history and QT interval changes when treating patients with haloperidol [4], [9], [11]. QT prolongation by haloperidol is ascribed to its blocking effects on the cardiac potassium channel hERG and was shown in various clinical studies [12], [13], [14], [15]. Most clinical studies were performed in selected healthy populations, or in sick patients with high intravenous doses [6], [16]. In clinical practice, however, haloperidol is mostly prescribed orally at low doses to elderly patients with multiple co-morbidities. Thus, clinicians are often faced with the need to prescribe haloperidol to patients with other potential causes of QT prolongation, including concomitant medication use and cardiac pathology (e.g., heart failure). Yet, studies on QT prolongation during low-dose haloperidol use in such high-risk patients are lacking.

In this study, we examined whether there is an association between haloperidol use and QT duration changes in a common population of elderly hospitalized patients with multiple morbidities and co-medications. By analyzing the ECGs of these patients before, during and after haloperidol use, we studied whether QT duration changed during the in-hospital use of haloperidol, taking other clinical factors into account. Also, by comparing patient characteristics and (acute) medical condition before haloperidol use, we aimed to define patient groups at risk for dangerous QT prolongation during haloperidol use. In particular, given that hospitalized elderly patients are often given haloperidol because they are agitated during an acute phase response, after surgery, or during acute hospitalization [17], [18], we studied whether these factors may impact on a patient's QTc response to haloperidol.

## Methods

### Study population and design

In this retrospective cohort study, we used a database of all 1788 in-hospital patients for whom haloperidol was prescribed between 2005 and 2007 during admission at the Academic Medical Centre, a university teaching hospital in Amsterdam. We analyzed all patients (N = 237) of whom three ECG recordings were made before, during and after haloperidol prescription, i.e., i) between two weeks and one day before start of haloperidol, ii) between one hour after start of haloperidol and 24 h after the last dose of haloperidol, and iii) >one week after the last dose of haloperidol [19], [20].

The study was conducted according to the principles expressed in the Declaration of Helsinki. All data were retrieved from the hospital's databases (where data of routine clinical care measures are stored) and were analysed anonymously. The medical ethics committee of the Academic Medical Center (Amsterdam) waived the need for approval and informed consent.

### Measurements

ECG analysis was conducted while the researchers were blinded for haloperidol status. QT durations were measured by hand and corrected for heart rate using Bazett's formula (QTc). All three measurements (before-haloperidol ECG, during-haloperidol ECG, and after-haloperidol ECG) were analyzed in the same lead for each patient, based on the best readable recording. In case of atrial fibrillation or irregular heart rates, the mean of all RR intervals in the recording was used for rate correction. Patients who had ECGs with multiple ventricular extrasystoles, pacemaker beats, left or right bundle branch block, or whose QTc duration could not be reliably measured (typically due to flat T waves) were excluded from further analysis. Of each patient, we obtained the following information from the hospital records: gender, age, medical history, medical status during hospital admission before haloperidol use (e.g., reason of admission, any surgery for which general anesthesia was given within 3 days before haloperidol use), actual administration and given dose of haloperidol, and the use of other QT prolonging drugs (CERT list 1 [21]) within the 72 hours before each ECG recording. We collected data of electrolytes measured within 48 hour before or after each ECG recording, using the measurement closest to the moment of recording, or within 24 hours of the recording moment in the case of serum potassium values. Signs of an acute phase response or inflammation at the time of each ECG recording were defined as either body temperature >39°C, or serum CRP level >100 mg/l, or leukocyte count >100*10E9/L. To analyze whether the QTc duration before haloperidol use influenced the levels of change in QTc duration, we stratified the patients into three subgroups of before-haloperidol QTc duration, based on the European Society of Cardiology Guidelines [22]: 1) Normal (male ≤430 ms, female ≤450 ms), 2) Borderline (male 431–450 ms, female 451–470 ms), and 3) Abnormal (male >450 ms, female >470 ms).

### Data analysis

Statistical Package for the Social Sciences (SPSS, version 16.0 for Mac) was used for data analysis. Changes in mean QTc duration before, during and after haloperidol use, and differences in the change of QTc duration per subgroup were tested for significance using a mixed model analysis assuming fixed effects, thereby accounting for changes in relevant clinical factors. We evaluated associations with patient variables, haloperidol dose, co-medication, medical history, medical status during hospital admission, electrolyte levels, and ECG parameters. Factors that were univariately associated (p<0.25) with before-haloperidol QTc duration, change in QT duration, or subgroup stratification were entered in the mixed model analysis. The final model estimated changes in QTc duration while accounting for relevant variables (backward selection with p<0.05). To test the possibility that an above normal QRS width influenced observed QTc duration, we performed a second analysis in which we did not use the measured QTc duration as the dependent variable, but the QTc duration minus excess QRS duration (i.e., QRS width minus 110 ms).

Risk factors for potentially dangerous QTc prolongation were analyzed using logistic regression in SPSS. Potentially dangerous QTc prolongation was defined as an increase in QTc duration by >50 ms or to >500 ms, or the occurrence of Torsade de Pointes [23]. Factors that were associated with a p<0.05 in the univariate analyses were entered into the multivariate models. Effect sizes were expressed in odds ratios (OR) with their corresponding 95% confidence intervals (CI) and p-values. Data are expressed as mean±standard deviation (SD), unless otherwise indicated.

## Results

**Figure 1:**
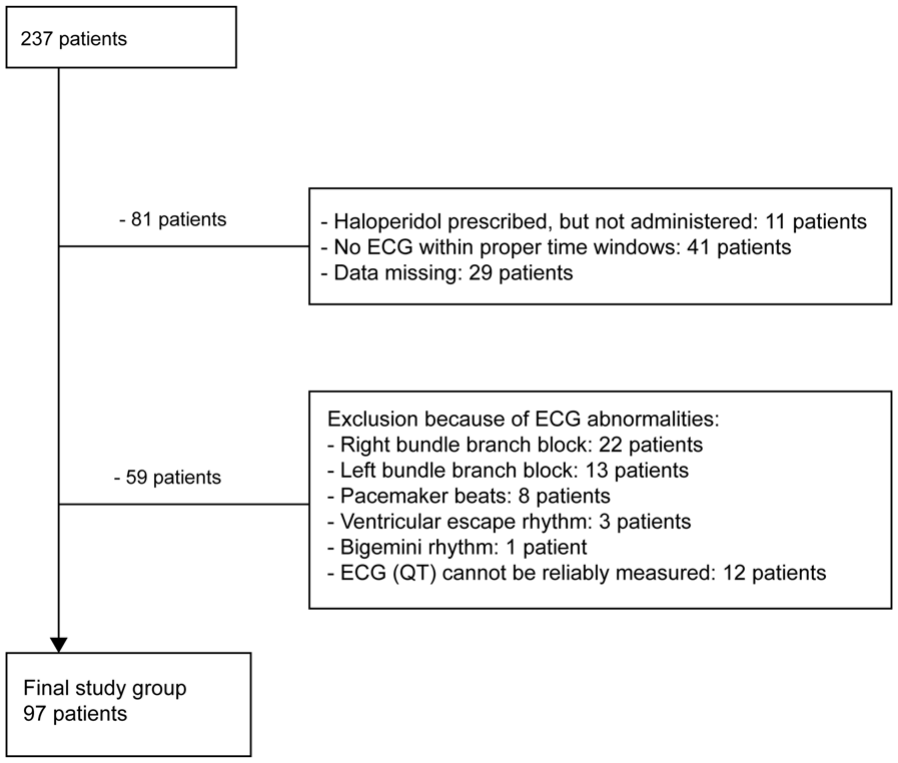
Flowchart of patient inclusion.

From a total cohort of 1788 patients for whom haloperidol was prescribed between 2005 and 2007 (mean age 67 years, male 59%), we first extracted all patients of whom an ECG recorded before, during, and after haloperidol prescription was available; this yielded 237 patients (mean age 70 years, male 66%). Since the prescription of haloperidol often reads ‘when necessary’, we verified in the patient's medical charts whether haloperidol was actually administered, and whether the ECG was recorded during actual haloperidol use. We excluded 81 patients of whom we could not establish this (Figure 1). We further excluded 59 patients because of ECG abnormalities that hindered a valid and reliable measurement of QT duration, yielding a study population of 97 patients for final analysis (mean age 68 years, male 64%). In case of missing data of serum levels of sodium or potassium, we imputed the mean value.

Table 1 presents baseline characteristics of the study population. Thirty to 41% of patients in the three subgroups underwent surgery and 70–78% had signs of inflammation. Subgroup stratification by QTc duration before haloperidol use was significantly associated with gender (more males in abnormal group, p = 0.002) and QRS interval (p = 0.049), but with none of the other measured conditions. Dose of haloperidol was 2,6 mg per day on average, (range: 0.5–10 mg per day), and was given orally in 75% of patients and intravenously in 25% of patients.

**Table 1:** Baseline characteristics of patients: all, and stratified in subgroups based on QTc duration before haloperidol.

	All patients (N = 97)	Normal (N = 57)	Borderline (N = 17)	Abnormal (N = 23)
Male, n (%)*	62 (63.9)	29 (50.9)	12 (70.6)	21 (91.3)
Age, years, mean (SD)	67.7 (14.3)	68.2 (14.5)	66.1 (15.8)	67.4 (13.5)
Dose of haloperidol, mean (SD)	2.6 (2.2)	2.5 (2.3)	2.3 (1.9)	3.1 (2.1)
QTc duration, ms, mean (SD)*	433.6 (43.2)	408.8 (24.6)	444.4 (11.3)	487.0 (43.0)
Heart rate, beats per minute, mean (SD)	86.8 (25.6)	85.0 (23.9)	98.0 (31.0)	83.1 (24.0)
QRS width, ms, mean (SD)	93.2 (15.1)	91.3 (14.9)	91.8 (16.2)	99.0 (13.7)
Serum sodium level, mmol/l, mean (SD)	139.8 (3.6)	139.5 (3.8)	140.5 (3.9)	140.0 (2.9)
Serum potassium level, mmol/l, mean (SD)	4.1 (0.5)	4.1 (0.4)	4.3 (0.5)	3.9 (0.6)
Diabetes, n (%)	20 (20.6)	12 (21.1)	4 (23.5)	4 (17.4)
Hypertension, n (%)	41 (42.3)	22 (38.6)	9 (53.9)	10 (43.5)
Heart failure, n (%)	11 (11.3)	5 (8.8)	3 (17.6)	3 (13.0)
Ischemic heart disease, n (%)	54 (55.7)	31 (54.4)	9 (52.9)	14 (60.9)
Surgery before haloperidol use, n (%)	36 (37.1)	22 (38.6)	7 (41.2)	7 (30.4)
Signs of inflammation, n (%)	70 (72.2)	40 (70.2)	12 (78.3)	18 (78.3)
Use of QT interval prolonging drugs, n (%)	6 (6.2)	3 (5.3)	0 (0)	3 (13.3)

Normal QTc duration: male <430 ms, female <450 ms,; borderline QTc duration: male 431–450 ms, female 451–470 ms;

abnormal QTc duration: male >450 ms, female >470 ms.

*Significant differences between subgroups (p<0.05).

**Figure 2:**
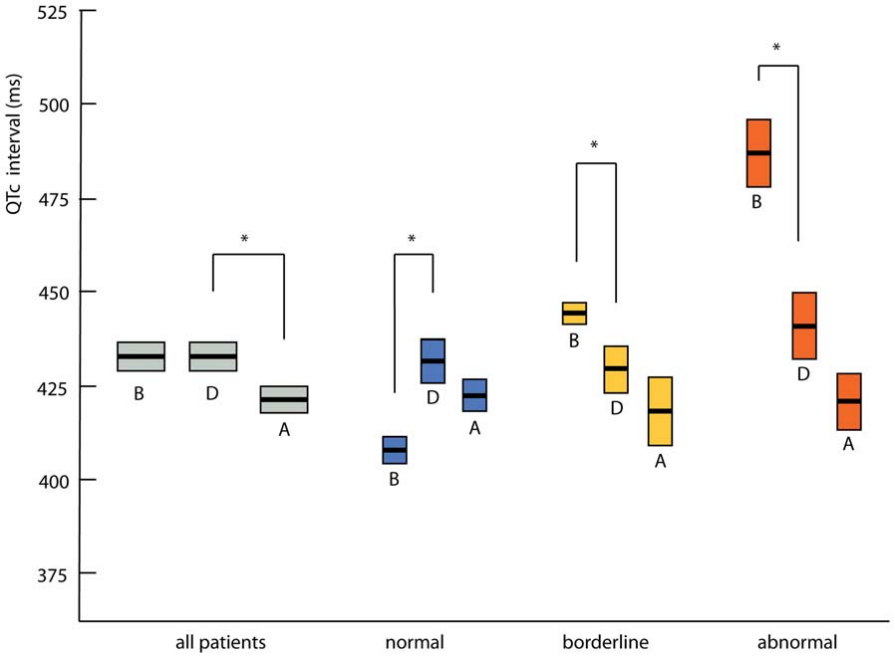
Course of changes in QTc duration before, during and after haloperidol use. Mean QTc intervals before, during and after haloperidol use of total patient group and of each subgroup, with Standard Errors of the mean. Normal: male ≤430 ms, female ≤450 ms, borderline: male 431–450 ms, female 451–470 ms, abnormal: male >450 ms, female >470 ms. B: before haloperidol use, D: during haloperidol use, A: after haloperidol use. * indicates significant change at p<0.05 level.

When we studied mean QTc duration of our overall study group, we found no change in QTc duration upon haloperidol use (Figure 2) and a decrease (−12.1±48.9 ms [p = 0.017]) after discontinuation of haloperidol. When we analyzed subgroups stratified by before-haloperidol QTc duration (normal, borderline and abnormal), we found that the normal group had significant QTc prolongation during haloperidol use (mean increase 23.1±45.5 ms [p<0.001], Figure 1). Twenty-three percent of these patients rose to abnormal levels. In contrast, the subgroups with borderline and abnormal before-haloperidol QTc duration showed significant QTc shortening upon haloperidol use (borderline: −14.6±26.2 ms [p = 0.035], and abnormal: −45.9±55.5 ms [p = 0.001]). Twenty-three percent of patients in the borderline group, and nine percent of patients in the abnormal group obtained abnormal levels. After discontinuation of haloperidol, all three groups showed a decrease in QTc duration (normal: −9.1±47.7 ms [p = 0.156]; borderline: −11.4±43.9 ms [p = 0.298]; abnormal: −20.0±56.0 ms [p = 0.101]).

Differences in QTc duration were found significant in the final mixed models analysis when comparing QTc durations before and during the use of haloperidol in the normal and abnormal subgroups, after adjusting for relevant covariates (signs of infection and surgery before haloperidol use). Changes in QTc duration after haloperidol discontinuation were not significant. Overall, the course of the changes in QTc duration was significantly different for each subgroup (p<0.001). Repeating the analysis with QRS-duration subtracted from the measured QTc duration did not yield different results.

In total, 16 patients (16%) developed potentially dangerous QTc prolongation during haloperidol use, 15 (94%) of whom had a normal before-haloperidol QTc duration. In only 2 patients QTc duration increased to >500 ms. No episodes of Torsade de Pointes were found.

We analyzed factors associated with dangerous QTc prolongation (Table 2). Five risk factors showed significant ORs in the univariate analysis (gender, before-haloperidol QTc duration, heart rate, surgery before haloperidol use, and signs of inflammation before haloperidol use), and were subsequently entered in the multivariate analysis. ORs and their corresponding 95% confidence intervals are presented in Table 2. Surgery before haloperidol use was strongly associated with increased risk of potentially dangerous QTc prolongation (OR 34.9 [p = 0.009]), as was male gender (OR 7.6 [p = 0.055]), though the latter not statistically significantly. Longer before-haloperidol QTc duration (OR 0.94 [p = 0.004]), faster before-haloperidol heart rate (OR 0.9 [p = 0.029]) and signs of inflammation (OR 0.1 [p = 0.147]) were associated with reduced risk of QTc prolongation, though not statistically significant for inflammation. In contrast, dose of haloperidol, mode of administration, and presence of cardiovascular risk factors were not associated with potentially dangerous QTc interval prolongation (only heart failure and ischemic heart disease increased the risk, though not statistically significantly). The model estimated 90.7% of the cases correctly.

**Table 2:** Unadjusted and adjusted Odds Ratios for potentially dangerous QTc prolongation.

Variable	Potentially dangerous QTc prolongation		Univariate			Multivariate I*		
	Yes (n = 16)	No (n = 81)	OR	95% CI	p-value	OR*	95% CI	p-value
Male, n (%)	14 (87.5)	48 (59.3)	4.8	1.0–22.6	0.046	7.6	1.0–60.5	0.055
Age years, mean (SD)	70.5 (9.7)	67.1 (15.1)	1.0	1.0–1.1	0.380			
Dose of haloperidol, mg, mean (SD)	2.4 (2.2)	2.6 (2.2)	0.9	0.7–1.2	0.682			
Before-haloperidol QTc, ms, mean (SD)	401.8 (24.4)	439.8 (43.4)	0.97	0.95–0.99	0.001	0.94	0.91–0.98	0.004
Normal QTc, n (%)**	15 (26.3)	42 (73.7)						
Borderline QTc, n (%)**	0 (0.0)	17 (100)	n.a.					
Abnormal QTc, n (%)**	1 (4.3)	22 (95.7)	0.1	0.0–1.0	0.053			
Heart rate, beats per min., mean (SD)	68.4 (15.3)	90.5 (25.7)	0.9	0.9–1.0	0.004	0.9	0.8–1.0	0.029
QRS width, ms, mean (SD)	99.1 (14.9)	92.0 (14.9)	1.0	1.0–1.1	0.090			
Serum sodium level, mmol/L, mean (SD)	139.9 (3.2)	139.7 (3.9)	1.0	0.9–1.2	0.860			
Serum potassium level, mmol/L, mean (SD)	4.1 (0.4)	4.1 (0.5)	0.9	0.3–2.7	0.876			
Diabetes, n (%)	2 (12.5)	14 (17.3)	0.5	0.1–2.4	0.387			
Hypertension, n (%)	6 (37.5)	35 (43.2)	0.8	0.3–2.4	0.673			
Heart failure, n (%)	3 (18.8)	8 (9.9)	2.1	0.5–9.0	0.315			
Ischemic heart disease, n (%)	10 (62.5)	44 (54.3)	1.4	0.5–4.2	0.548			
Surgery before haloperidol use, n (%)	13 (81.2)	23 (28.4)	10.9	2.8–41.9	<0.001	34.9	2.4–506.2	0.009
Signs of inflammation, n (%)	7 (43.8)	63 (77.8)	0.2	0.1–0.7	0.008	0.1	0.0–1.1	0.147
Use of QT interval prolonging drugs, n (%)	2 (12.5)	4 (4.9)	2.7	0.5–16.5	0.268			

OR: Odds Ratio, CI: confidence interval, SD: Standard Deviation, n.a.: not applicable.

*Multivariate I: ORs in the multivariate analysis are adjusted for gender, baseline QTc duration in ms, heart rate, surgery before haloperidol, and signs of inflammation.

## Discussion

In this real-life study among hospitalized elderly patients with multiple co-morbidities, we found that substantial QTc prolongation upon low-dose haloperidol use occurred predominantly in patients with normal before-haloperidol QTc duration. Conversely, in most patients with borderline or abnormal before-haloperidol QTc duration, an unexpected QTc shortening occurred. Importantly, 94% of patients with potentially dangerous QTc prolongation had a before-haloperidol QTc duration in the normal range. A rise in QTc duration to potentially dangerous levels was not only associated with before-haloperidol QTc duration, but also with surgery (increased risk) and signs of inflammation (decreased risk) before haloperidol use. Although patients with cardiovascular risk factors are reported to have a higher risk of QTc prolongation [24], [25], we did not find significant associations in our study population.

It is unlikely that QTc shortening in patients with a prolonged QTc interval prior to haloperidol use is due to the effects of haloperidol *per se*. A more plausible explanation might be that changes in the underlying condition occurred during haloperidol use, and that these changes caused QTc duration to shorten, despite haloperidol use. For instance, although speculative, it is possible that the complex effects of acute illness (both cardiac and non-cardiac), acute phase response, or poor general condition (e.g., post-operative patients), contributed to QTc prolongation through as yet unknown mechanisms. In support of the view that acute illness may cause QTc prolongation, average QTc durations of all three subgroups were normal after haloperidol use, i.e., when the acute illness had subsided.

Unexpectedly, potentially dangerous QTc-prolongation was associated with absence of signs of inflammation, though not significantly. We expected the contrary since inflammation/fever is suggested as a risk factor for QTc prolongation in patients with a decreased hERG function (e.g., patients using QT prolonging drugs) [17], [26]. Proposed mechanisms include the effects of circulating inflammatory cytokines on ionic currents that determine QT duration, and increased drug-mediated block of hERG channels at high temperature, as demonstrated for erythromycin [27], [28]. It is conceivable that inflammation/fever and haloperidol may act in an additive manner in blocking the hERG channel, thus causing clinically dangerous QTc prolongation. Yet, we observed that signs of inflammation decreased the risk for potentially dangerous QTc prolongation. Thus, the presence of inflammation before haloperidol use was not associated with potentially dangerous QTc prolongation during haloperidol use. One possible explanation may be that the QT prolonging effect of inflammation in these patients was stronger than that of haloperidol. Thus, attenuation of the inflammation between the before-haloperidol state and the during-haloperidol state may have been associated with attenuation of the QT prolonging effects of inflammation (i.e., QT shortening); this effect may be stronger than the QT prolonging effect of haloperidol; the net result would be QT shortening (or lack of further QT prolongation).

A limitation of our study is that the patients had different causes and stages of inflammation or acute phase response; thus, different levels and types of circulating cytokines may have been present. Therefore, it remains difficult to link the changes in QT duration during haloperidol use directly to the effects of inflammation. Moreover, the indication for haloperidol prescription (e.g., agitation) may also be associated with QTc prolongation, independent of haloperidol use. This may be the case in post-operative patients, who, in our population, are at increased risk to develop potentially dangerous QTc prolongation. A stress response has been shown to induce QTc prolongation [29], which may, at least in part, explain our findings. Further research is needed to establish whether the various indications for haloperidol prescription in elderly hospitalized patients are associated with changes in QTc duration, and to elucidate the underlying mechanisms of these changes.

To our knowledge, this is the first study that attempts to evaluate changes in QTc duration upon haloperidol use, while taking co-morbidities that play an important role in a real-life clinical setting into account. A major limitation of our study, however, is its retrospective design. By limiting our study to patients of whom ECG-recordings were available before, during and after haloperidol use, we excluded patients who were discharged or died during haloperidol use. Moreover, we were biased towards patients in whom multiple ECG recordings were made, thereby probably introducing an overrepresentation of patients with cardiac disease. This might explain the large proportion of male patients in our study population.

Our findings would seem to suggest that ECG monitoring of elderly patients who receive in-hospital low-dose haloperidol may not be necessary, as QTc prolongation occurred mostly in patients with normal before-haloperidol QTc duration, while QTc duration generally shortened in those with before-haloperidol QTc prolongation. Overall, potentially dangerous QTc prolongation occurred only in few patients, and Torsade de Pointes was recorded in none. Yet, we see the largely surprising findings of our study (in particular, the differential effects between subgroups) as indication that the real-life clinical effects of haloperidol are not well-established. We therefore conclude that more research (larger prospective studies) is needed to allow for rational clinical decision making on the cardiac safe use of haloperidol.
