# Correction: Differential Changes in QTc Duration during In-Hospital Haloperidol Use

**DOI:** 10.1371/annotation/92655d2b-fac1-4641-970c-a8e4fb5739fc

**Published:** 2012-07-10

**Authors:** Marieke T. Blom, Abdennasser Bardai, Barbara C. van Munster, Mei-Ing Nieuwland, Hendrik de Jong, Daniel A. van Hoeijen, Anne M. Spanjaart, Anthonius de Boer, Sophia E. de Rooij, Hanno L. Tan

There was an error in the published funding statement. The correct funding statement is: H.L. Tan was supported by the Netherlands Organization for Scientific Research (NWO, grant ZonMW Vici 918.86.616), the Dutch Medicines Evaluation Board (MEB/CBG), and the European Community's Seventh Framework Programme (FP7/2007-2013) under grant agreement nr. 241679 - the ARITMO project. A. Bardai was supported by the Netherlands Organization for Scientific Research (NWO, grant Mozaiek 017.003.084). Both grants are unrestricted. The funders had no role in study design, data collection and analysis, decision to publish, or preparation of the manuscript. 

